# Supplementary material for: Hippo pathway controls biopterin metabolism to shield adjacent cells from ferroptosis in lung cancer
Source: EMBO Rep. 2025 Jul 7;26(16):4124–52. doi: 10.1038/s44319-025-00515-4 (PMC12373837; doi:10.1038/s44319-025-00515-4)
Supplement: Supplementary file 1 — Table EV1 [file 44319_2025_515_MOESM1_ESM.docx]

**Table EV1. Clinicopathological factors in patients with lung adenocarcinoma.**

The association between YAP/TAZ expression patterns and clinicopathological factors in patients with lung adenocarcinoma was described.

|  |  | YAP/TAZ expression pattern | | |
| --- | --- | --- | --- | --- |
|  |  | Heterogenous | Homogenous | p |
| Age |  |  |  |  |
|  | <65 | 12 | 44 | 0.713 |
|  | ≥65 | 34 | 105 |  |
| Gender |  |  |  |  |
|  | Male | 29 | 60 | 0.0106 |
|  | Female | 17 | 89 |  |
| Smoking |  |  |  |  |
|  | B.I.* <600 | 23 | 107 | 0.00755 |
|  | B.I.* ≥600 | 23 | 42 |  |
| Pathological stage |  |  |  |  |
|  | 0-I | 17 | 110 | 0.000013 |
|  | II-VI | 29 | 39 |  |
| Nuclear grade |  |  |  |  |
|  | 1,2 | 39 | 145 | 0.00415 |
|  | 3 | 7 | 4 |  |
| EGFR mutation** |  |  |  |  |
|  | negative | 22 | 63 | 0.29 |
|  | positive | 19 | 81 |  |

Fisher’s exact test was performed. Underline indicates statistically significant.

*B.I., Brinkman Index

**Among 195 patient-derived samples, 10 patient samples were not tested for EGFR gene mutations. EGFR, epidermal growth factor receptor.
