# Supplementary material for: Hippo pathway controls biopterin metabolism to shield adjacent cells from ferroptosis in lung cancer
Source: EMBO Rep. 2025 Jul 7;26(16):4124–52. doi: 10.1038/s44319-025-00515-4 (PMC12373837; doi:10.1038/s44319-025-00515-4)
Supplement: Supplementary file 3 — Table EV3 [file 44319_2025_515_MOESM3_ESM.docx]

**Table EV3. MRM Parameters for Related Molecules.**

The multiple reaction monitoring (MRM) parameters used in the study are provided.

| Analyte | Precursor Ion (*m/z*) | Product Ion (*m/z*) | Fragmentor Voltage (V) | Collision Energy (eV) | Polarity |
| --- | --- | --- | --- | --- | --- |
| BH4 | 242.1 | 166.1 | 90 | 17 | + |
| BH2 | 240.1 | 196.1 | 90 | 9 | + |
| [^13^C_2_,^15^N]GS-HPE-AM | 488.2 | 359.1 | 130 | 13 | + |
| GS-HPE-AM | 485.2 | 356.1 | 152 | 13 | + |
