# Supplementary figures and images for: Hippo pathway controls biopterin metabolism to shield adjacent cells from ferroptosis in lung cancer
Source: EMBO Rep. 2025 Jul 7;26(16):4124–52. doi: 10.1038/s44319-025-00515-4 (PMC12373837; doi:10.1038/s44319-025-00515-4)

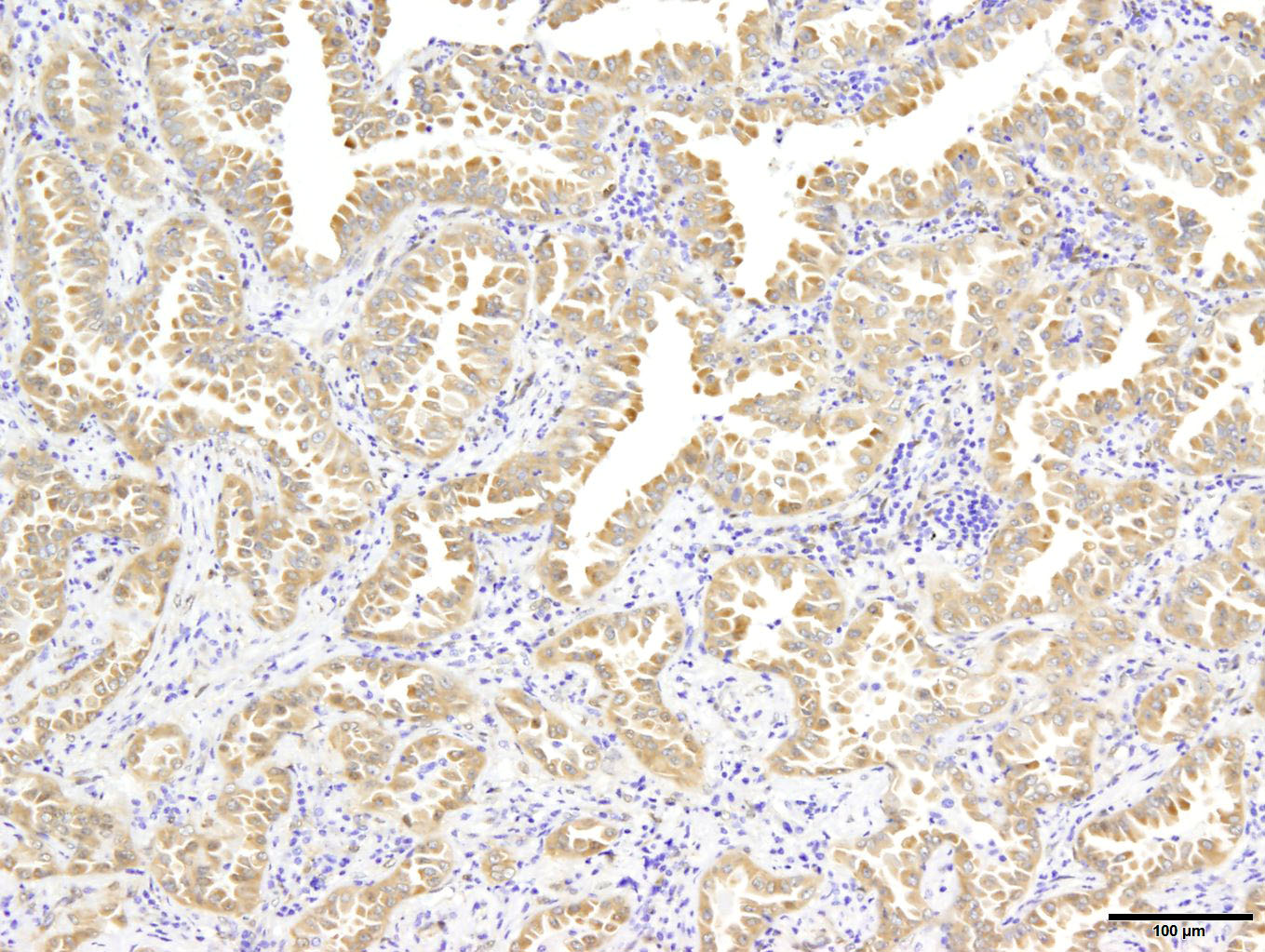

Supplement: Supplementary file 7 — Source data Fig. 1 [file 44319_2025_515_MOESM7_ESM.zip › Figure 1/1A/Diffuse.jpg]

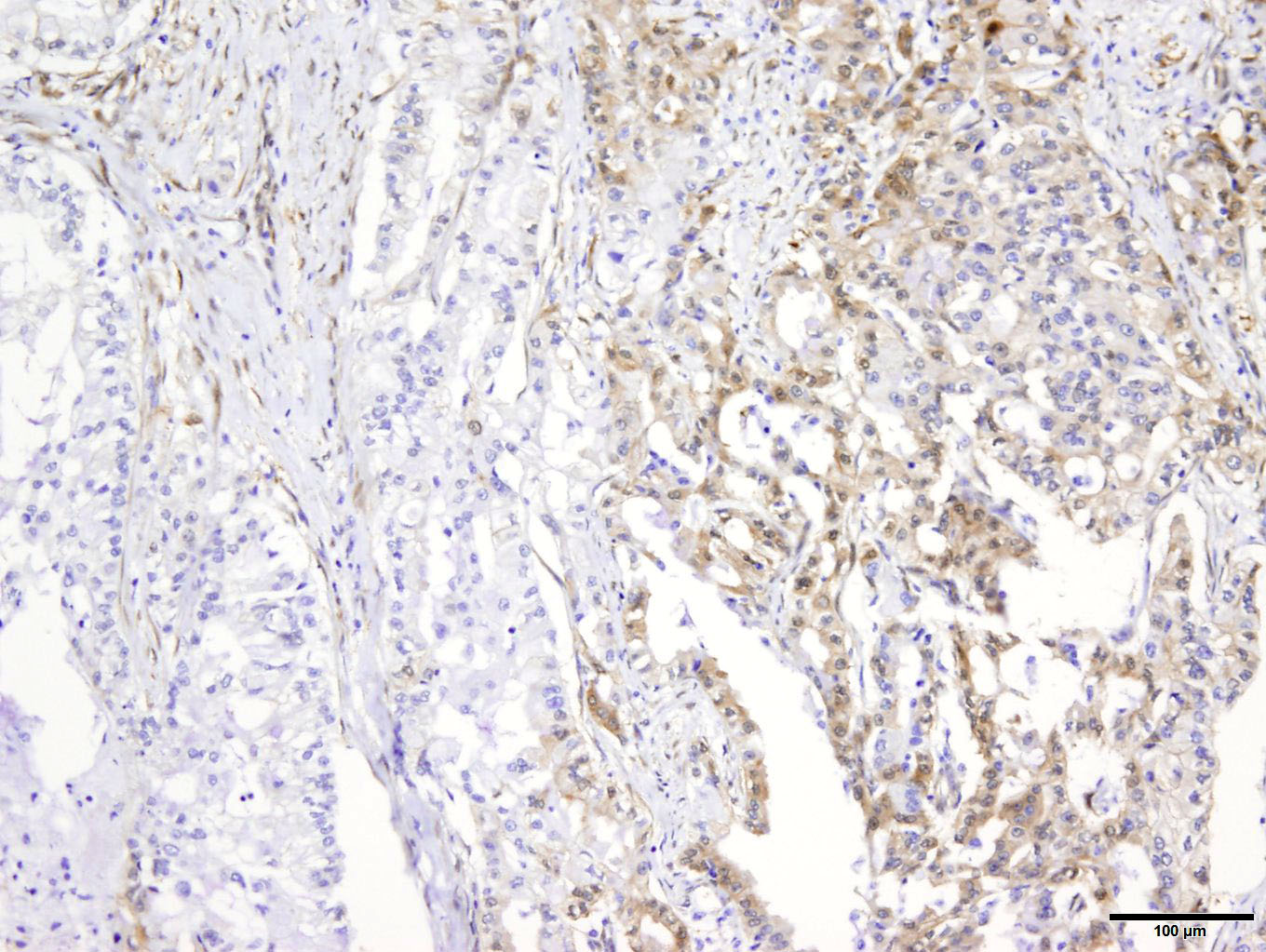

Supplement: Supplementary file 7 — Source data Fig. 1 [file 44319_2025_515_MOESM7_ESM.zip › Figure 1/1A/Hetero.jpg]

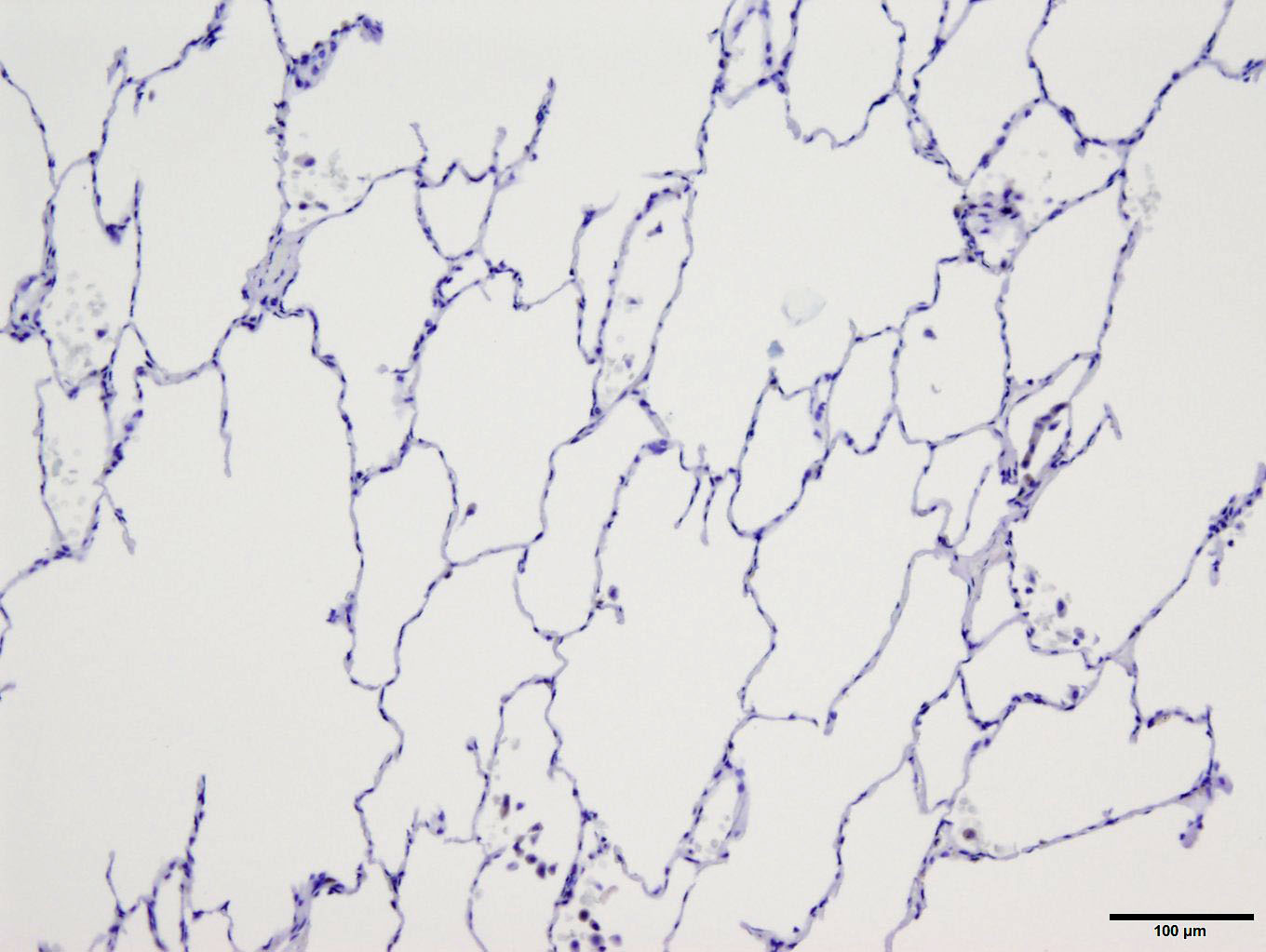

Supplement: Supplementary file 7 — Source data Fig. 1 [file 44319_2025_515_MOESM7_ESM.zip › Figure 1/1A/Normal lung.jpg]

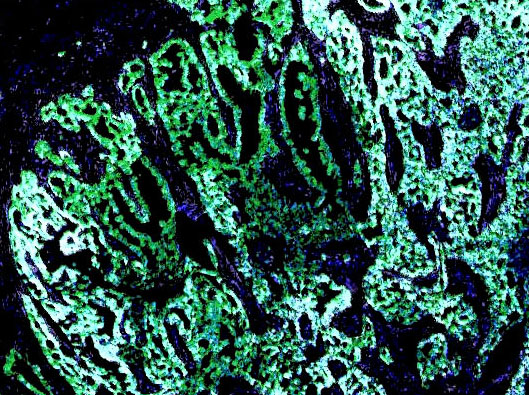

Supplement: Supplementary file 7 — Source data Fig. 1 [file 44319_2025_515_MOESM7_ESM.zip › Figure 1/1B/heterogeneous_fusion.jpg]

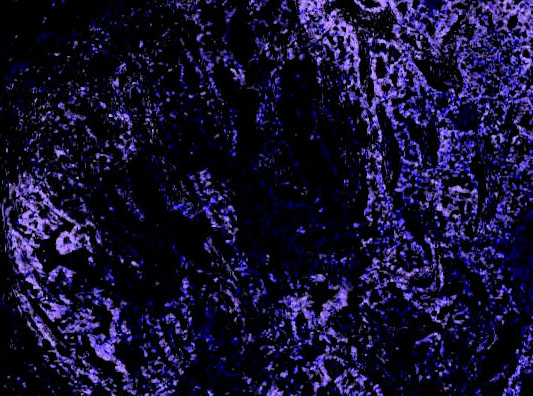

Supplement: Supplementary file 7 — Source data Fig. 1 [file 44319_2025_515_MOESM7_ESM.zip › Figure 1/1B/heterogeneous_YAP.jpg]

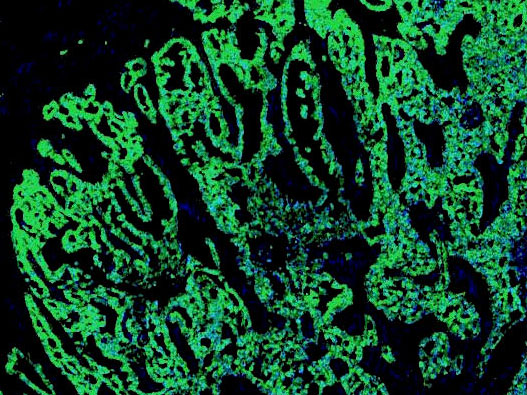

Supplement: Supplementary file 7 — Source data Fig. 1 [file 44319_2025_515_MOESM7_ESM.zip › Figure 1/1B/heterogeneous_CK.jpg]

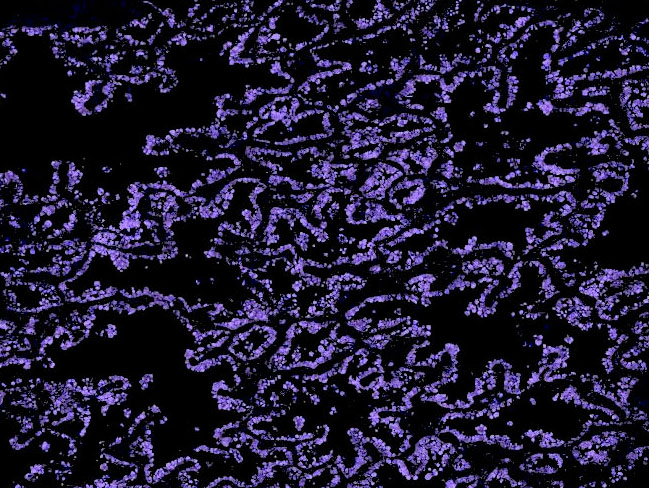

Supplement: Supplementary file 7 — Source data Fig. 1 [file 44319_2025_515_MOESM7_ESM.zip › Figure 1/1B/homogeneous_YAP.jpg]

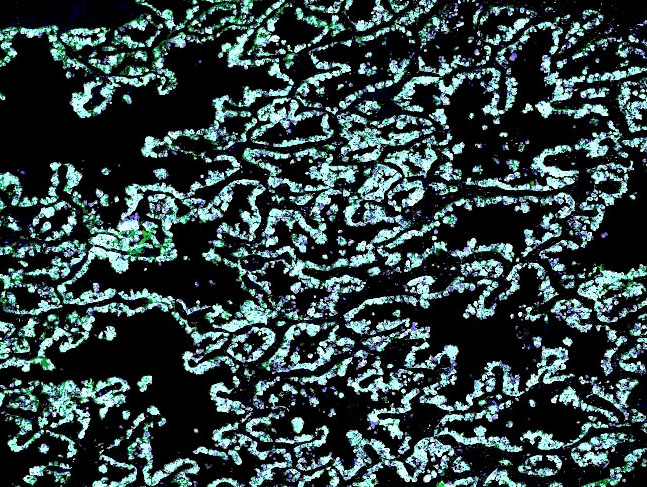

Supplement: Supplementary file 7 — Source data Fig. 1 [file 44319_2025_515_MOESM7_ESM.zip › Figure 1/1B/homogeneous_fusion.jpg]

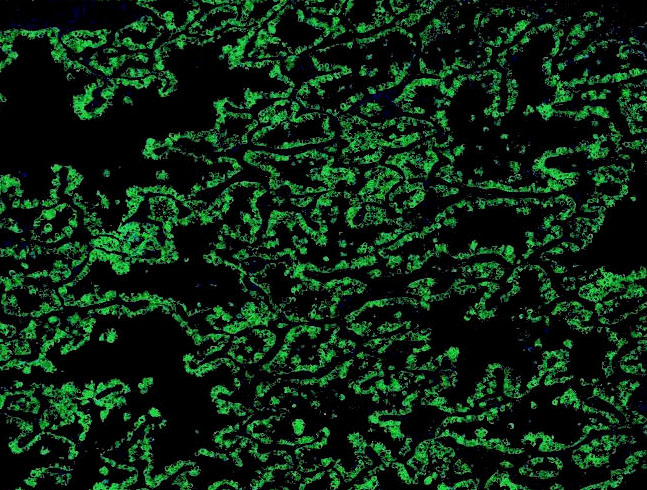

Supplement: Supplementary file 7 — Source data Fig. 1 [file 44319_2025_515_MOESM7_ESM.zip › Figure 1/1B/homogeneous_CK.jpg]

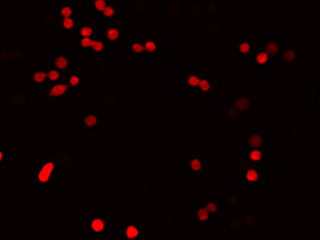

Supplement: Supplementary file 8 — Source data Fig. 2 [file 44319_2025_515_MOESM8_ESM.zip › Figure 2/2C/YAP:TAZ dKO-tdTomato RSL3 0h.tif]

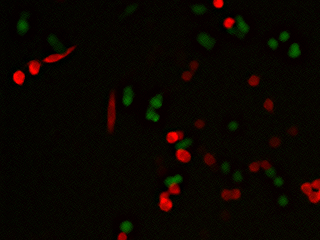

Supplement: Supplementary file 8 — Source data Fig. 2 [file 44319_2025_515_MOESM8_ESM.zip › Figure 2/2C/Mix culture RSL3 10h.tif]

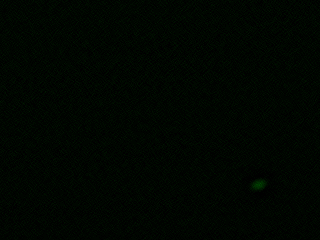

Supplement: Supplementary file 8 — Source data Fig. 2 [file 44319_2025_515_MOESM8_ESM.zip › Figure 2/2C/WT-EGFP RSL3 10h.tif]

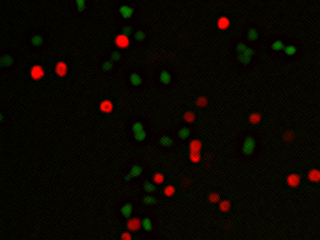

Supplement: Supplementary file 8 — Source data Fig. 2 [file 44319_2025_515_MOESM8_ESM.zip › Figure 2/2C/Mix culture RSL3 0h.tif]

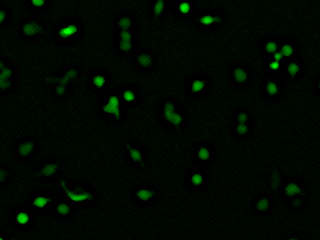

Supplement: Supplementary file 8 — Source data Fig. 2 [file 44319_2025_515_MOESM8_ESM.zip › Figure 2/2C/WT-EGFP RSL3 0h.tif]

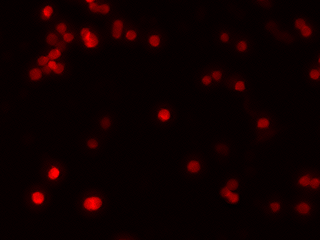

Supplement: Supplementary file 8 — Source data Fig. 2 [file 44319_2025_515_MOESM8_ESM.zip › Figure 2/2C/YAP:TAZ dKO-tdTomato RSL3 10h.tif]

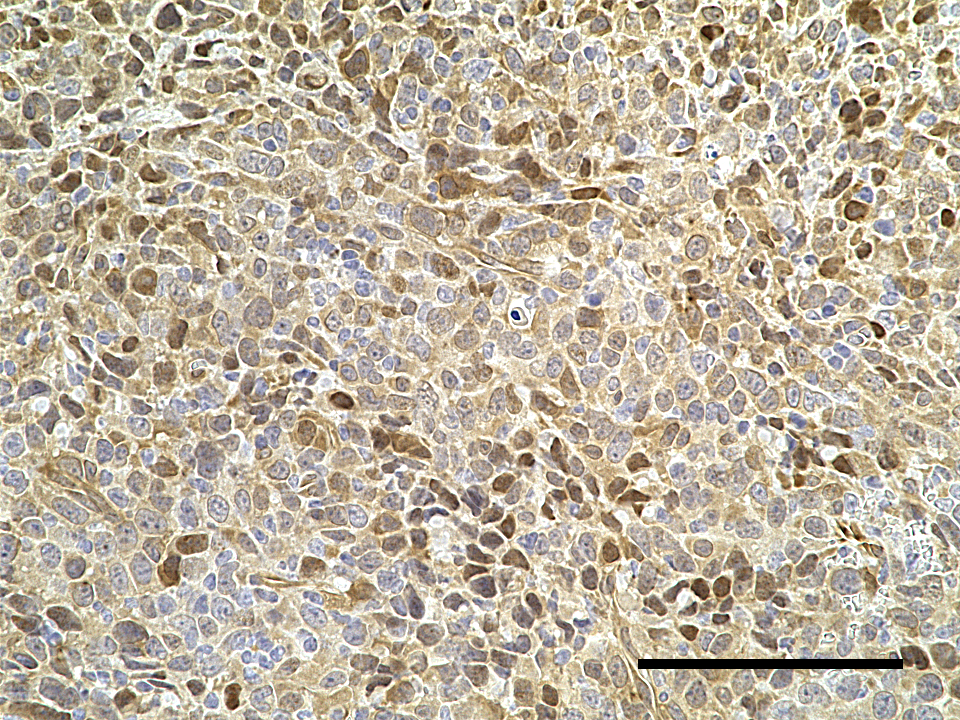

Supplement: Supplementary file 8 — Source data Fig. 2 [file 44319_2025_515_MOESM8_ESM.zip › Figure 2/2B/Mouse YAPTAZ.tif]

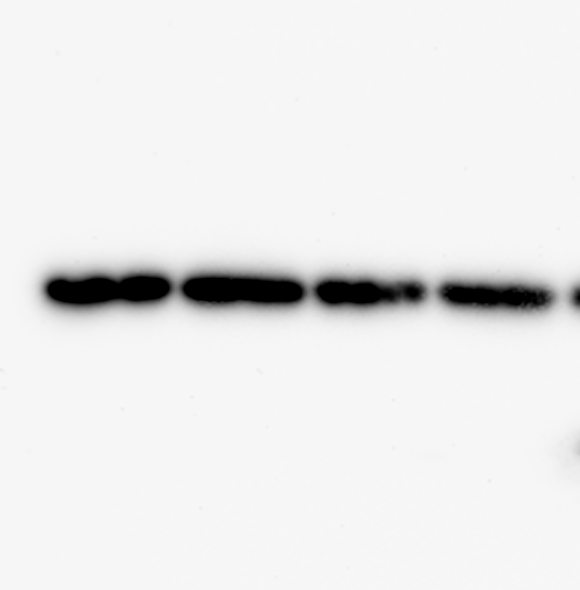

Supplement: Supplementary file 9 — Source data Fig. 3 [file 44319_2025_515_MOESM9_ESM.zip › Figure 3/3E/western Actin.tif]

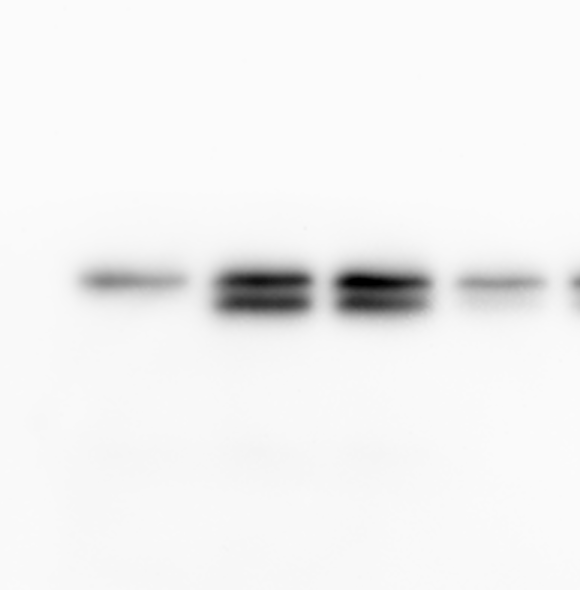

Supplement: Supplementary file 9 — Source data Fig. 3 [file 44319_2025_515_MOESM9_ESM.zip › Figure 3/3E/western GCH1.tif]

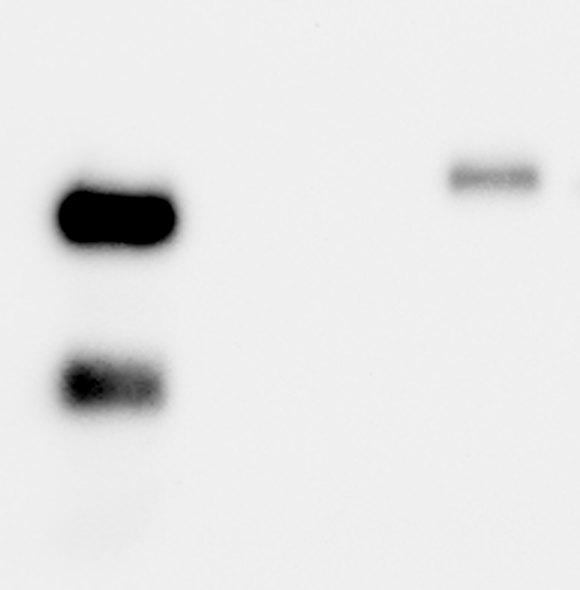

Supplement: Supplementary file 9 — Source data Fig. 3 [file 44319_2025_515_MOESM9_ESM.zip › Figure 3/3E/western YAP:TAZ.tif]

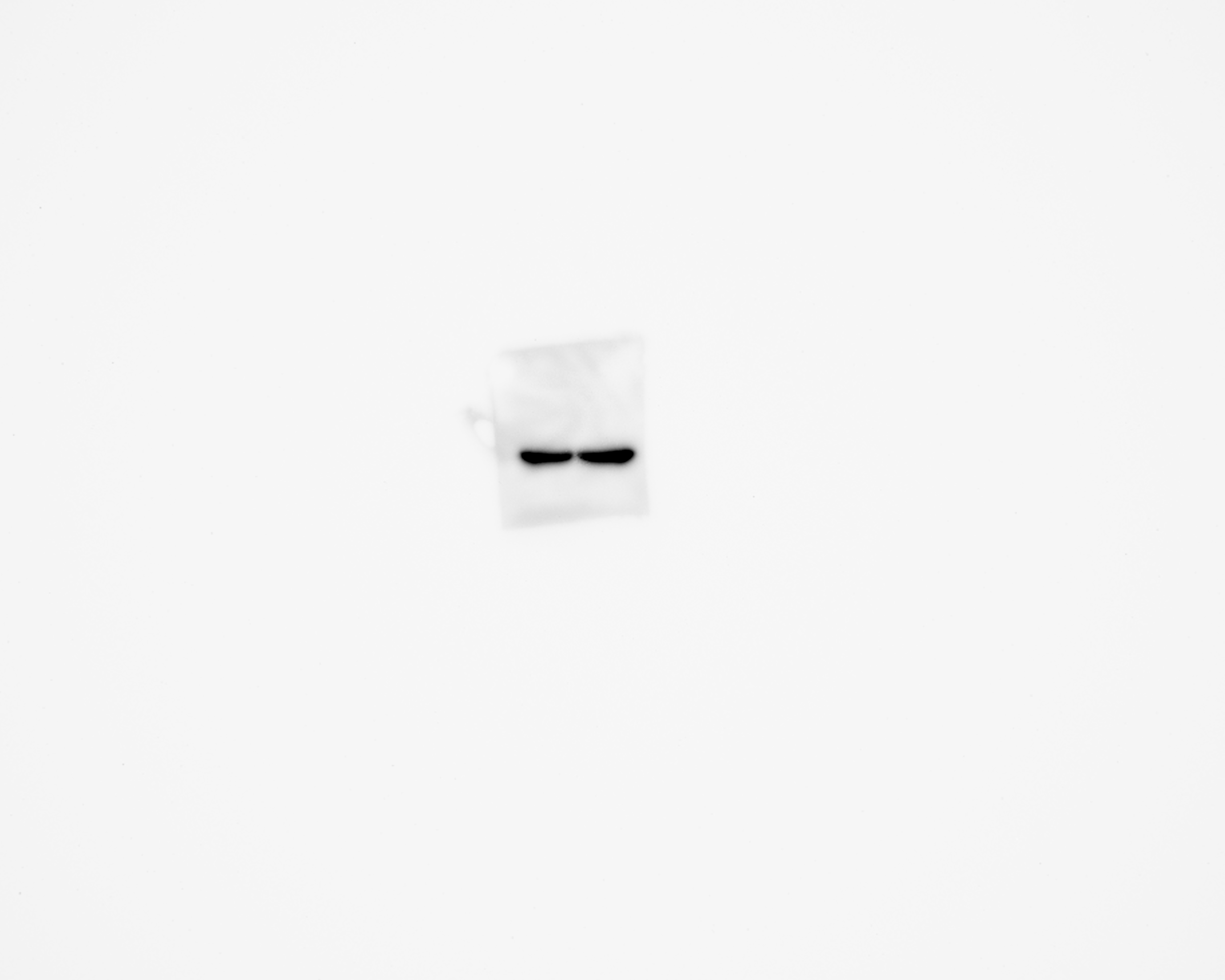

Supplement: Supplementary file 9 — Source data Fig. 3 [file 44319_2025_515_MOESM9_ESM.zip › Figure 3/3B/western Actin.tif]

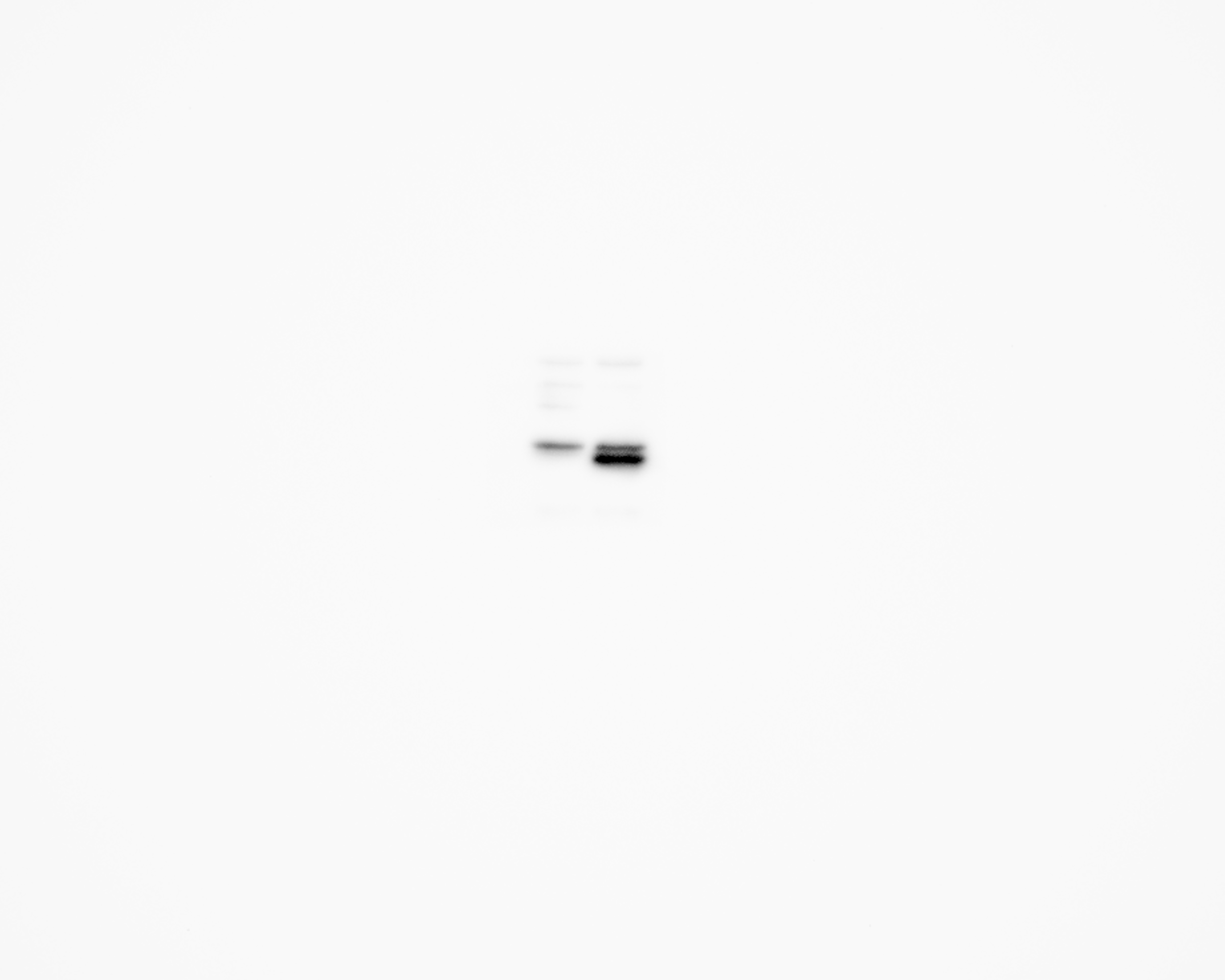

Supplement: Supplementary file 9 — Source data Fig. 3 [file 44319_2025_515_MOESM9_ESM.zip › Figure 3/3B/western GCH1.tif]

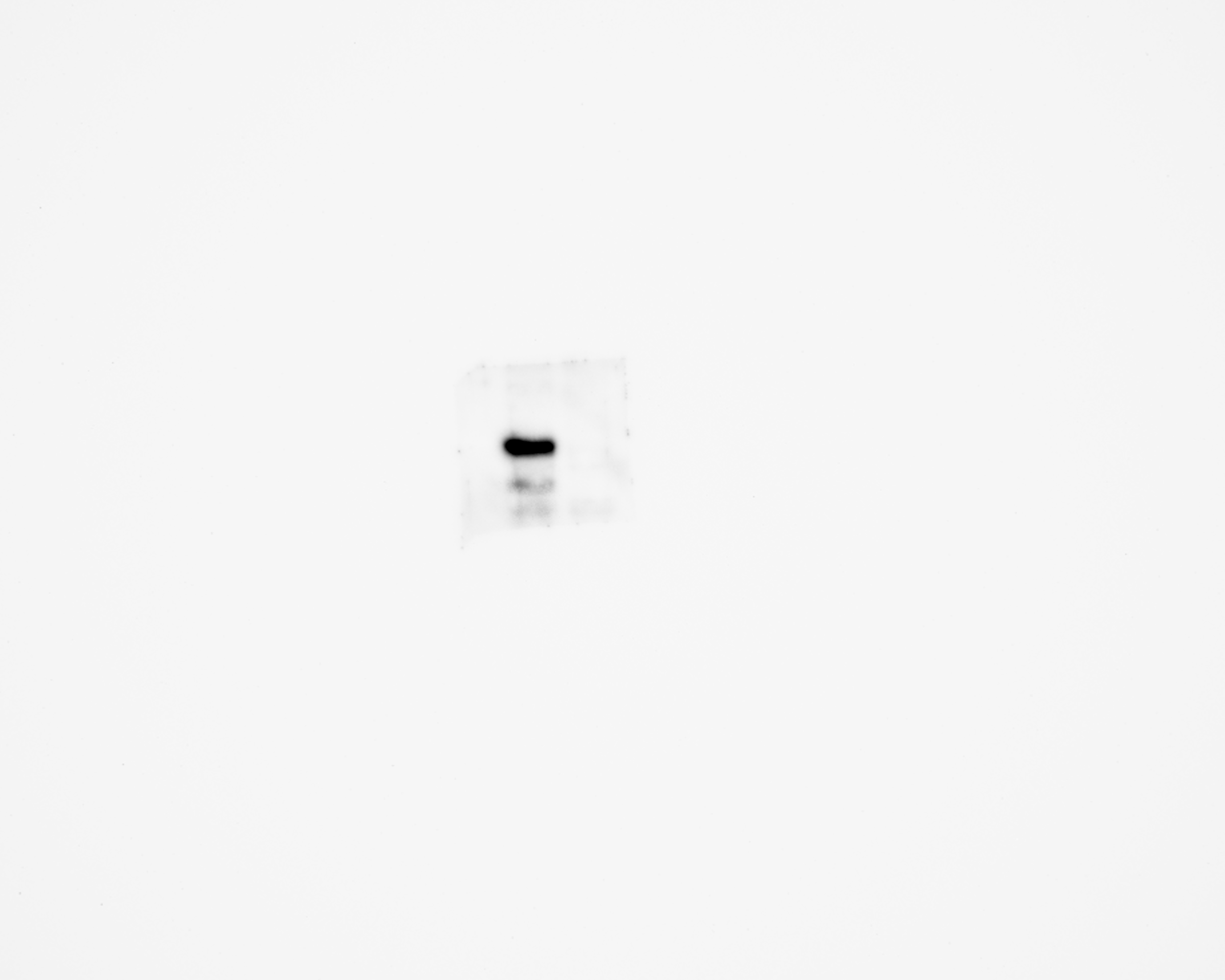

Supplement: Supplementary file 9 — Source data Fig. 3 [file 44319_2025_515_MOESM9_ESM.zip › Figure 3/3B/western YAP:TAZ.tif]

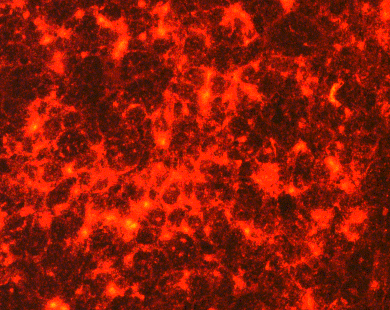

Supplement: Supplementary file 9 — Source data Fig. 3 [file 44319_2025_515_MOESM9_ESM.zip › Figure 3/3D/YAP:TAZ dKO.tif]

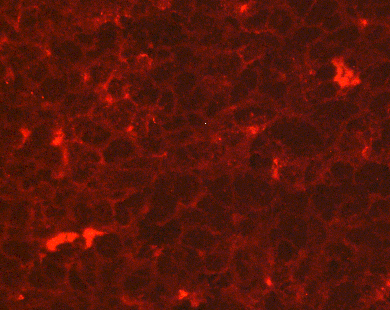

Supplement: Supplementary file 9 — Source data Fig. 3 [file 44319_2025_515_MOESM9_ESM.zip › Figure 3/3D/WT.tif]

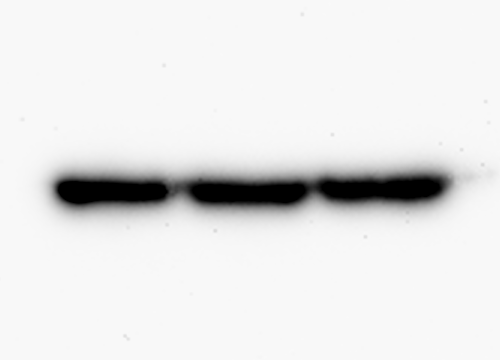

Supplement: Supplementary file 10 — Source data Fig. 4 [file 44319_2025_515_MOESM10_ESM.zip › Figure 4/4C/western Actin.tif]

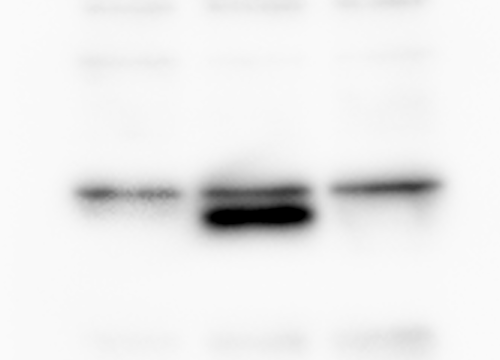

Supplement: Supplementary file 10 — Source data Fig. 4 [file 44319_2025_515_MOESM10_ESM.zip › Figure 4/4C/western GCH1.tif]

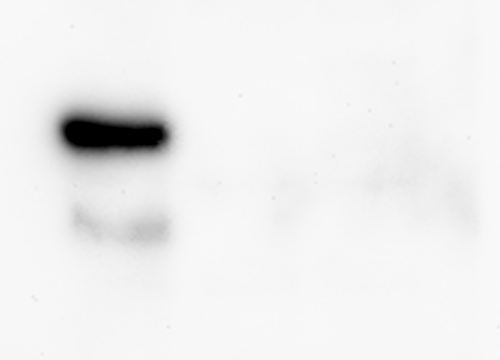

Supplement: Supplementary file 10 — Source data Fig. 4 [file 44319_2025_515_MOESM10_ESM.zip › Figure 4/4C/western YAP:TAZ.tif]

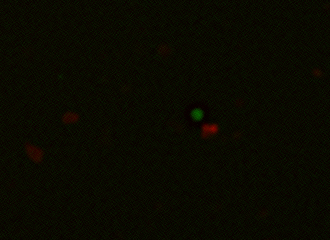

Supplement: Supplementary file 10 — Source data Fig. 4 [file 44319_2025_515_MOESM10_ESM.zip › Figure 4/4F/Mix culture RSL3 10h.tif]

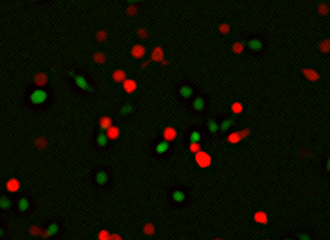

Supplement: Supplementary file 10 — Source data Fig. 4 [file 44319_2025_515_MOESM10_ESM.zip › Figure 4/4F/Mix culture RSL3 0h.tif]
