# Supplementary material for: Hippo pathway controls biopterin metabolism to shield adjacent cells from ferroptosis in lung cancer
Source: EMBO Rep. 2025 Jul 7;26(16):4124–52. doi: 10.1038/s44319-025-00515-4 (PMC12373837; doi:10.1038/s44319-025-00515-4)
Supplement: Supplementary file 12 — Expanded View Figures [file 44319_2025_515_MOESM12_ESM.pdf]

Expanded View Figures

Figure EV1. Generating yes-associated protein (YAP)/transcriptional co-activator with PDZ-binding motif (TAZ) (YAP/TAZ)-deficient Lewis lung carcinoma (LLC) cells.

(**A**) DNA mutation in YAP/TAZ double-knockout (dKO) LLC cells using the clustered regularly interspaced short palindromic repeat (CRISPR)/Cas9 system. gDNA, genomic DNA. (**B**) Immunoblotting (IB) of cell extracts from wild-type (WT) and YAP/TAZ dKO LLC cells with antibodies against the indicated proteins. (**C**) Reverse transcription (RT) and real-time PCR analysis of the YAP/TAZ target gene *Ankrd1* in WT and YAP/TAZ dKO LLC cells. Data are means ± SD of three biologically independent samples from a representative experiment. ****$P = 0.000001244429960$ (unpaired t test).

 © The Author(s)

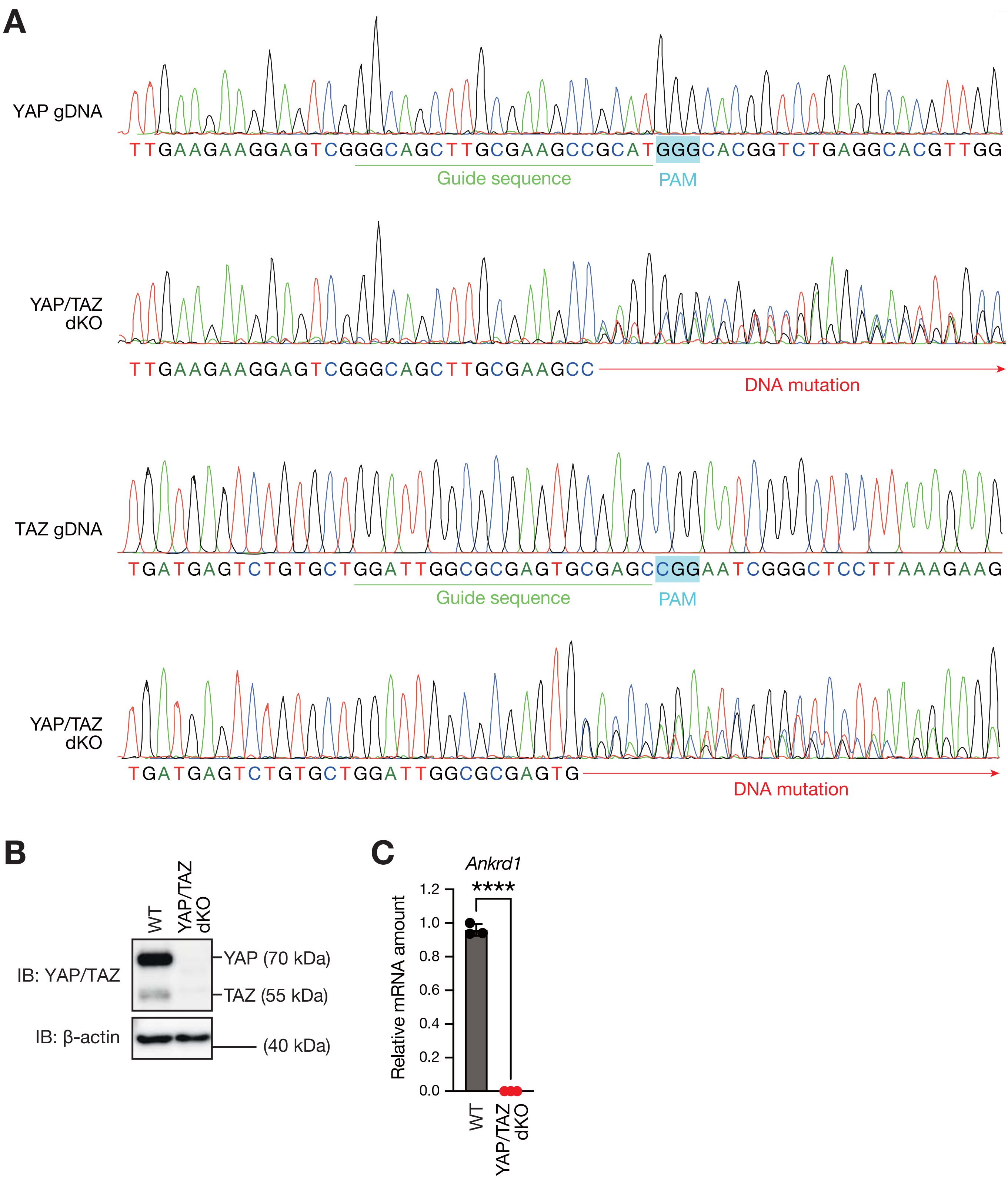
A
YAP gDNA
TTGAAGAAGGAGTCGGGCAGCTTGCGAAGCCGCATGGGCACGGTCTGAGGCACGTTGG
Guide sequence
PAM
YAP/TAZ dKO
TTGAAGAAGGAGTCGGGCAGCTTGCGAAGCC
DNA mutation
TAZ gDNA
TGATGAGTCTGTGCTGGATTGGCGCGAGTGCGAGCCGGAATCGGGCTCCTTAAAGAAG
Guide sequence
PAM
YAP/TAZ dKO
TGATGAGTCTGTGCTGGATTGGCGCGAGTG
DNA mutation
B
WT
YAP/TAZ dKO
IB: YAP/TAZ
YAP (70 kDa)
TAZ (55 kDa)
IB: β-actin
(40 kDa)
C
Ankrd1
****
Relative mRNA amount
1.2
1.0
0.8
0.6
0.4
0.2
0.0
WT
YAP/TAZ dKO

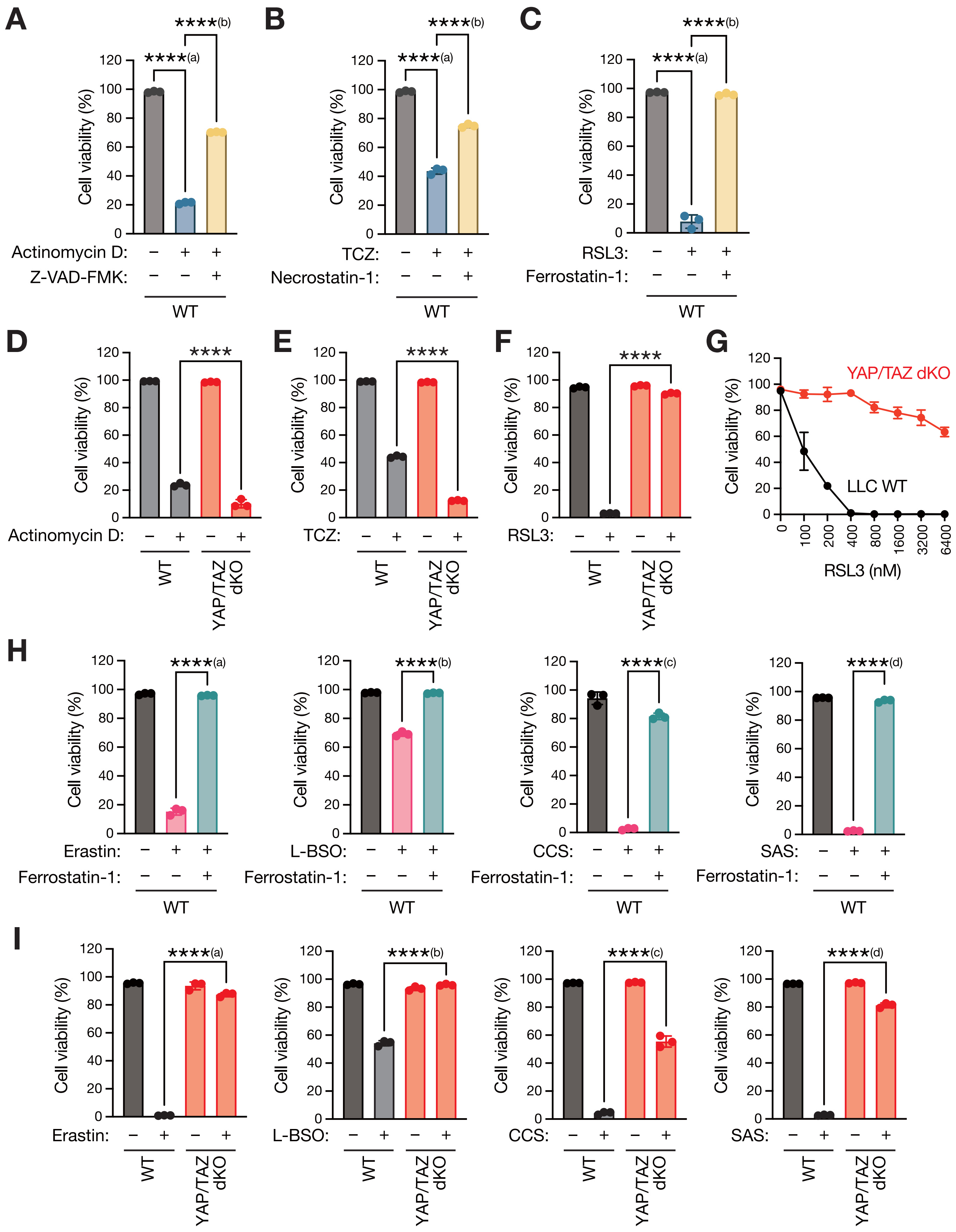
A
B
C
D
E
F
G
H
I
Cell viability (%)
****(a)
****(b)
****(c)
****(d)
****
Actinomycin D:
Z-VAD-FMK:
TCZ:
Necrostatin-1:
RSL3:
Ferrostatin-1:
WT
YAP/TAZ dKO
LLC WT
RSL3 (nM)
Erastin:
L-BSO:
CCS:
SAS:

Figure EV2. YAP/TAZ-deficient LLC cells were resistant to ferroptosis.

(**A**) Actinomycin D-induced cell death was rescued by the apoptosis inhibitor Z-VAD-FMK. Wild-type (WT) LLC cells were pretreated (or not) with Z-VAD-FMK (50 μM) for 1 h, followed by stimulation with Actinomycin D (500 nM) for 24 h. Dead cells were stained with propidium iodide (PI), and the percentage of the PI-negative live cell population was calculated using the flow cytometer. Data are means ± SD of three biologically independent samples from a representative experiment. ****(a)$P = 0.000000000000065$; ****(b)$P = 0.000000000000065$ (one-way ANOVA test followed by Tukey's multiple comparison test). (**B**) TCZ-induced cell death was rescued by the necroptosis inhibitor Necrostatin-1. WT LLC cells were pretreated (or not) with Necrostatin-1 for 1 h, followed by stimulation with TCZ [T, TNFα (30 ng/mL); C, cycloheximide (30 μg/mL); Z, Z-VAD-FMK (20 μM)] for 24 h. The percentage of live cell population was calculated using PI and flow cytometer. Data are means ± SD of three biologically independent samples from a representative experiment. ****(a)$P = 0.000000012723845$; ****(b)$P = 0.000000720360306$ (one-way ANOVA test followed by Tukey's multiple comparison test). (**C**) RSL3-induced cell death was rescued by the ferroptosis inhibitor Ferrostatin-1. WT LLC cells were pretreated (or not) with Ferrostatin-1 (5 μM) for 30 min, followed by stimulation with RSL3 (400 nM) for 10 h. The percentage of live cell population was calculated using PI and flow cytometer. Data are means ± SD of three biologically independent samples from a representative experiment. ****(a)$P = 0.000000048729652$; ****(b)$P = 0.000000056548852$ (one-way ANOVA test followed by Tukey's multiple comparison test). (**D**) YAP/TAZ loss sensitizes LLC cells to apoptosis. WT and YAP/TAZ dKO LLC cells were treated with Actinomycin D (500 nM) for 24 h, followed by cell viability assay using flow cytometer. Data are means ± SD of three biologically independent samples from a representative experiment. ****$P = 0.000030539048461$ (one-way ANOVA test followed by Tukey's multiple comparison test). (**E**) YAP/TAZ loss sensitizes LLC cells to necroptosis. WT and YAP/TAZ dKO LLC cells were treated with TCZ [T, TNFα (30 ng/mL); C, cycloheximide (30 μg/mL); Z, Z-VAD-FMK (20 μM)] for 24 h, followed by cell viability assay using flow cytometer. Data are means ± SD of three biologically independent samples from a representative experiment. ****$P = 0.000000000000054$ (one-way ANOVA test followed by Tukey's multiple comparison test). (**F**) YAP/TAZ loss confers resistance to ferroptosis in LLC cells. WT and YAP/TAZ dKO LLC cells were treated with RSL3 (400 nM) for 10 h, followed by cell viability assay using flow cytometer. Data are means ± SD of three biologically independent samples from a representative experiment. ****$P = 0.000000000000051$ (one-way ANOVA test followed by Tukey's multiple comparison test). (**G**) Cell viability analysis was performed on WT and YAP/TAZ dKO LLC cells treated with indicated concentrations of RSL3 for 10 h, followed by cell viability assay using flow cytometer. Data are means ± SD of three biologically independent samples from a representative experiment. (**H**) Cell death induced by ferroptosis-inducing reagents or cysteine and cystine starvation (CCS) was rescued by the ferroptosis inhibitor Ferrostatin-1. WT LLC cells were pretreated (or not) with Ferrostatin-1 (5 μM) for 30 min, followed by stimulation with Erastin (5 μM) for 10 h, L-Buthionine-(S,R)-Sulfoximine (L-BSO; 10 mM) for 24 h, or Sulfasalazine (SAS; 1 mM) for 24 h. For CCS, cells were cultured in the cysteine and cystine-starved medium for 24 h. The percentage of live cell population was calculated using flow cytometer. Data are means ± SD of three biologically independent samples from a representative experiment. ****(a)$P = 0.000000000005959$; ****(b)$P = 0.000000096903963$; ****(c)$P = 0.000000191080058$; ****(d)$P = 0.000000000000065$ (one-way ANOVA test followed by Tukey's multiple comparison test). (**I**) YAP/TAZ loss confers resistance to ferroptosis in LLC cells. WT and YAP/TAZ dKO LLC cells were treated with Erastin (5 μM) for 10 h, L-BSO (10 mM) for 24 h, or SAS (1 mM) for 24 h. For CCS, cells were cultured in the cysteine and cystine-starved medium for 24 h. The percentage of live cell population was calculated using flow cytometer. Data are means ± SD of three biologically independent samples from a representative experiment. ****(a)$P = 0.000000000000769$; ****(b)$P = 0.000000000883037$; ****(c)$P = 0.000000000024749$; ****(d)$P = 0.000000000000051$ (one-way ANOVA test followed by Tukey's multiple comparison test).

© The Author(s)

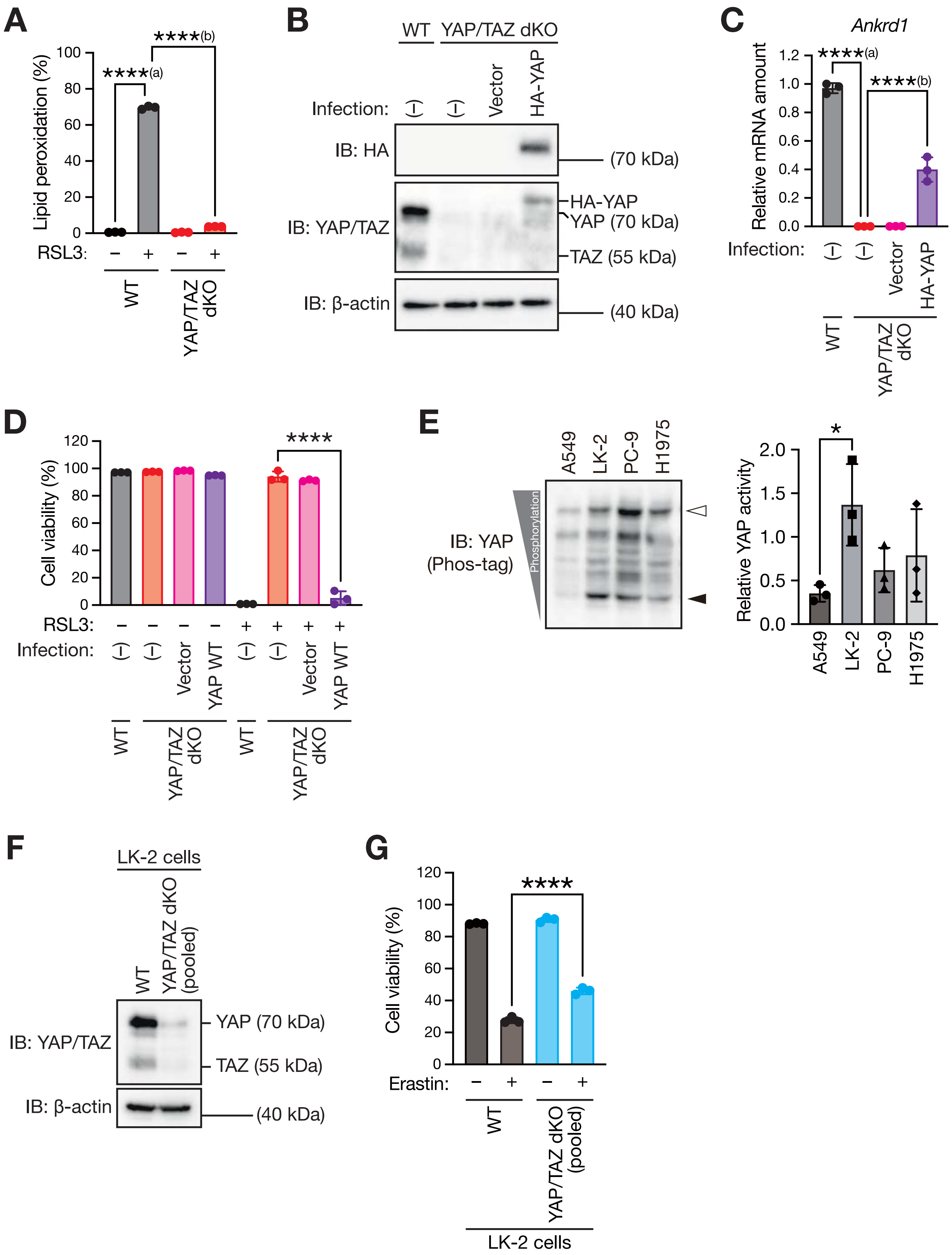
A
Lipid peroxidation (%)
****(a)
****(b)
RSL3:
WT
YAP/TAZ dKO
B
WT
YAP/TAZ dKO
Infection:
(-)
Vector
HA-YAP
IB: HA
(70 kDa)
IB: YAP/TAZ
HA-YAP
YAP (70 kDa)
TAZ (55 kDa)
IB: β-actin
(40 kDa)
C
Ankrd1
Relative mRNA amount
D
Cell viability (%)
****
YAP WT
E
A549
LK-2
PC-9
H1975
IB: YAP
(Phos-tag)
Phosphorylation
Relative YAP activity
F
LK-2 cells
YAP/TAZ dKO (pooled)
G
Erastin:

Figure EV3. YAP/TAZ determines the sensitivity to ferroptosis.

(**A**) YAP/TAZ depletion suppresses lipid peroxidation in LLC cells. WT and YAP/TAZ dKO LLC cells were treated with RSL3 (400 nM) for 4 h, followed by lipid peroxidation measurement using flow cytometer. Data are means ± SD of three biologically independent samples from a representative experiment. ****[(a)]$P = 0.000000000000051$; ****[(b)]$P = 0.000000000000051$ (one-way ANOVA test followed by Tukey's multiple comparison test). (**B**) Immunoblot (IB) analysis of cell extracts from WT and YAP/TAZ dKO LLC cells infected (or not infected) with HA-YAP expression vectors. (**C**) RT and real-time PCR analysis of the YAP/TAZ target *Ankrd1* gene in WT and YAP/TAZ dKO LLC cells infected (or not infected) with HA-YAP expression vectors. Data are means ± SD of three biologically independent samples from a representative experiment. ****[(a)]$P = 0.000000023517185$; ****[(b)]$P = 0.000027011157926$ (one-way ANOVA test followed by Tukey's multiple comparison test). (**D**) Re-expression of YAP sensitizes YAP/TAZ-deficient LLC cells to ferroptosis. WT and YAP/TAZ dKO LLC cells infected (or not) with expression vectors for HA-YAP were treated with RSL3 (400 nM) for 10 h, followed by cell viability assay using flow cytometer. Data are means ± SD of three biologically independent samples from a representative experiment. ****$P = 0.000000004002113$ (one-way ANOVA test followed by Tukey's multiple comparison test). (**E**) Left: Representative image of Phos-tag immunoblotting (IB) analysis of cell extracts from A549, LK-2, PC-9 and H1975 human lung cancer cells using antibodies against YAP. Right: The relative activity of YAP in each cell line was assessed based on the ratio of non-phosphorylated YAP (black arrowhead) to fully phosphorylated YAP (white arrowhead). Data are means ± SD of three biologically independent samples from a representative experiment. *$P = 0.044457726502518$ (one-way ANOVA test followed by Tukey's multiple comparison test). (**F**) Immunoblotting (IB) of cell extracts from WT and YAP/TAZ dKO LK-2 cells with antibodies against the indicated proteins. (**G**) WT and YAP/TAZ dKO LK-2 cells were treated with Erastin (5 μM) for 10 h, followed by cell viability assay using flow cytometer. Data are means ± SD of three biologically independent samples from a representative experiment. ****$P = 0.000002538628468$ (one-way ANOVA test followed by Tukey's multiple comparison test).

© The Author(s)

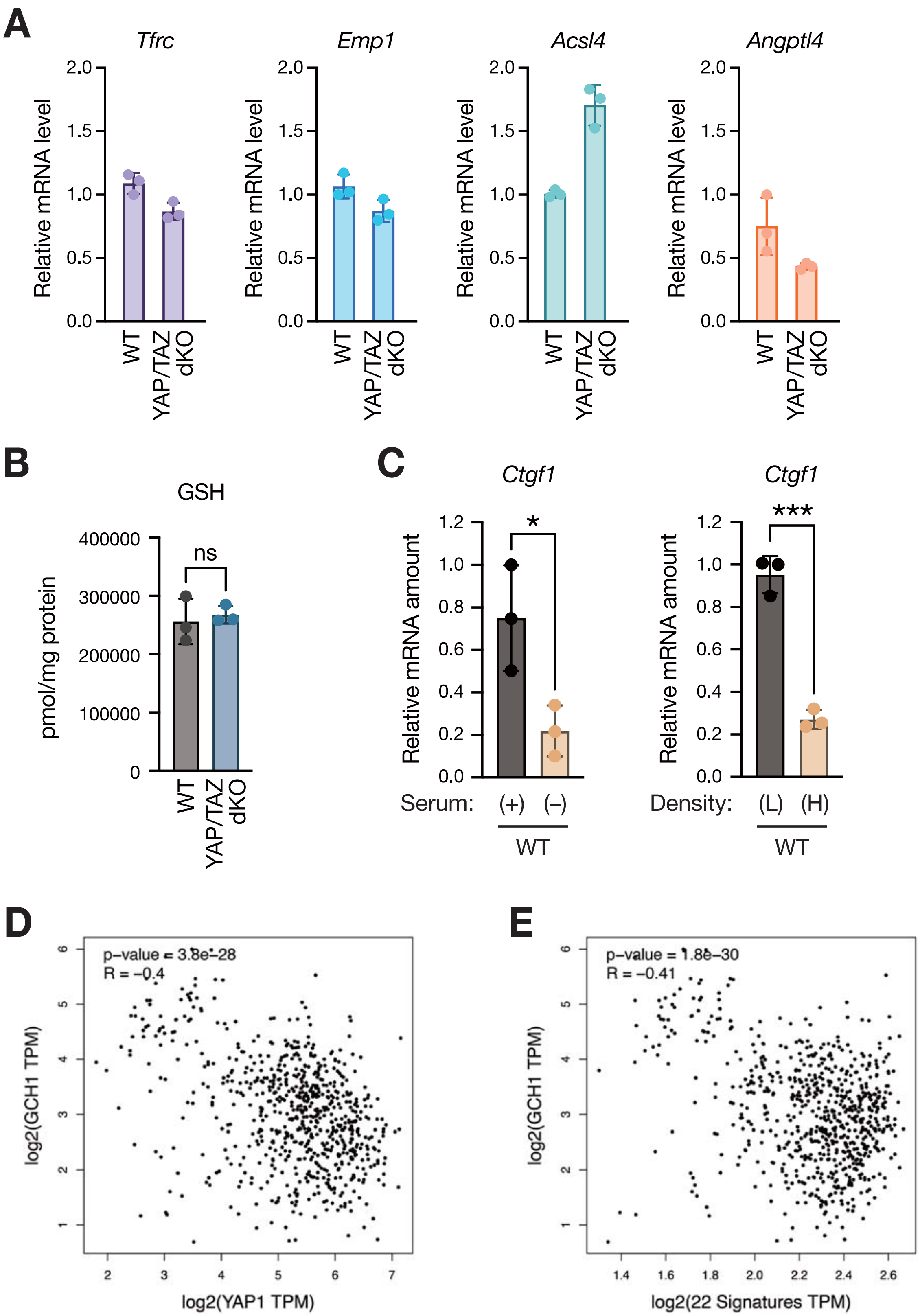
A
Tfrc
Emp1
Acsl4
Angptl4
Relative mRNA level
2.0
1.5
1.0
0.5
0.0
WT
YAP/TAZ dKO
B
GSH
pmol/mg protein
400000
300000
200000
100000
0
ns
WT
YAP/TAZ dKO
C
Ctgf1
Relative mRNA amount
1.2
1.0
0.8
0.6
0.4
0.2
0.0
*
***
Serum: (+) (–)
Density: (L) (H)
WT
D
p–value = 3.8e–28
R = –0.4
log2(GCH1 TPM)
log2(YAP1 TPM)
E
p–value = 1.8e–30
R = –0.41
log2(GCH1 TPM)
log2(22 Signatures TPM)

 © The Author(s)

Figure EV4. Impacts of YAP/TAZ inhibition in LLC cells.

(**A**) RT and real-time PCR analysis of the ferroptosis-related genes in WT and YAP/TAZ dKO LLC cells. Data are means ± SD of three biologically independent samples from a representative experiment. (**B**) Intracellular glutathione (GSH) levels were measured using LC-MS/MS. Data are means ± SD of three biologically independent samples from a representative experiment. ns indicates no significant difference, ns, not significant ($P = 0.658668395593572$) (unpaired t test). (**C**) RT and real-time PCR analysis of the YAP/TAZ target *Ctgf* gene in WT LLC cells cultured with or without serum for 32 h (left) or at low (L) or high (H) cell densities for 52 h (right). Data are means ± SD of three biologically independent samples from a representative experiment. *$P = 0.029089468282210$; ***$P = 0.000272320651963$ (unpaired t test). (**D**) The correlation between *GCH1* and *YAP1* mRNA levels across TCGA normal datasets was analyzed using the GEPIA (Gene Expression Profiling Interactive Analysis) tool with Pearson correlation. (**E**) The correlation between *GCH1* mRNA levels and the YAP/TAZ transcriptional target signature across TCGA normal datasets was analyzed using the GEPIA tool with Pearson correlation.

© The Author(s)

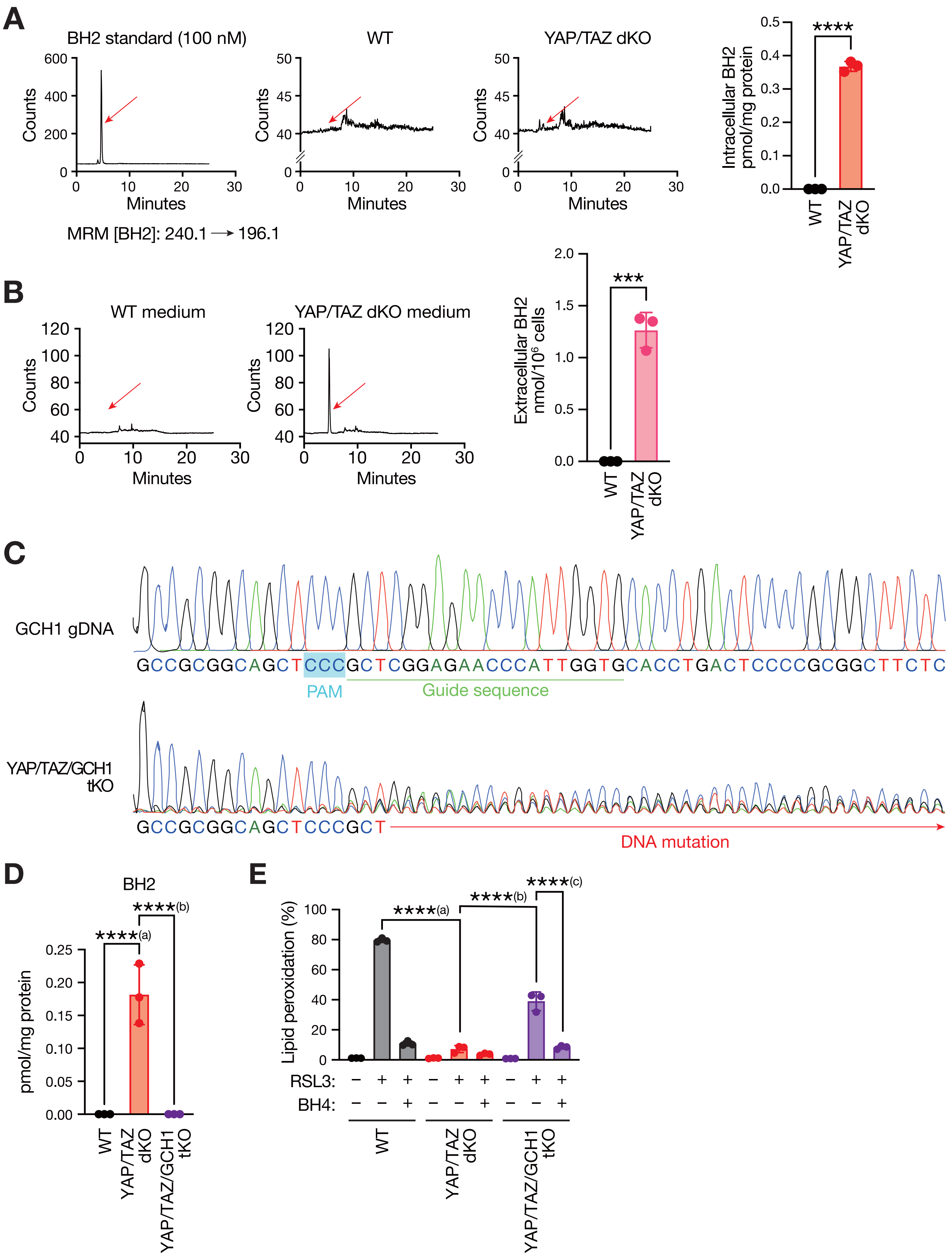
A
BH2 standard (100 nM)
WT
YAP/TAZ dKO
Counts
Minutes
MRM [BH2]: 240.1 → 196.1
Intracellular BH2 pmol/mg protein
****
WT
YAP/TAZ dKO
B
WT medium
YAP/TAZ dKO medium
Counts
Minutes
Extracellular BH2 nmol/10^6 cells
***
WT
YAP/TAZ dKO
C
GCH1 gDNA
GCCGCGGCAGCTCCCGCTCGGAGAACCCATTGGTGCACCTGACTCCCCGCGGCTTCTC
PAM
Guide sequence
YAP/TAZ/GCH1 tKO
GCCGCGGCAGCTCCCGCT
DNA mutation
D
BH2
pmol/mg protein
****(a)
****(b)
WT
YAP/TAZ dKO
YAP/TAZ/GCH1 tKO
E
Lipid peroxidation (%)
****(a)
****(b)
****(c)
RSL3:
BH4:
WT
YAP/TAZ dKO
YAP/TAZ/GCH1 tKO

 © The Author(s)

Figure EV5. GCH1 is required for ferroptosis resistance in YAP/TAZ-deficient LLC cells.

(**A**) Intracellular dihydrobiopterin (BH2) levels in WT and YAP/TAZ dKO LLC cells. Peaks with the corresponding multiple reaction monitoring (MRM) indicate intracellular BH2 (left, red arrows). Quantitative analysis of BH2 levels is shown on the right. Data are means ± SD of three biologically independent samples from a representative experiment. ****$P = 0.000001790642720$ (unpaired t test). (**B**) BH2 levels in the culture media from WT and YAP/TAZ dKO LLC cells. Peaks with the corresponding MRM indicate extracellular BH2 (red arrows). Quantitative analysis of extracellular BH2 levels is shown on the right. Data are means ± SD of three biologically independent samples from a representative experiment. ***$P = 0.000211344236570$ (unpaired t test). (**C**) DNA mutation in YAP/TAZ/GCH1 tKO LLC cells using the CRISPR/Cas9 system. gDNA, genomic DNA. (**D**) BH2 levels in WT, YAP/TAZ dKO, and YAP/TAZ/GCH1 tKO LLC cells. Data are means ± SD of three biologically independent samples from a representative experiment. ****[(a)]$P = 0.000357087917378$; ****[(b)]$P = 0.000357087917378$ (one-way ANOVA test followed by Tukey's multiple comparison test). (**E**) WT, YAP/TAZ dKO, and YAP/TAZ/GCH1 triple-knockout (tKO) LLC cells were pretreated (or not) with BH4 (40 μM) for 30 min, followed by stimulation with RSL3 (400 nM) for 4 h. The percentage of cells positive for lipid peroxidation was calculated using flow cytometer. Data are means ± SD of three biologically independent samples from a representative experiment. ****[(a)]$P = 0.000000000005554$; ****[(b)]$P = 0.000000155138171$; ****[(c)]$P = 0.000000230915225$ (one-way ANOVA test followed by Tukey's multiple comparison test).

© The Author(s)
